# Transcriptomic Analysis of the Liver Redox Response During Food-Anticipatory Activity Under a Time-Restricted Feeding Protocol in Rats

**DOI:** 10.3390/antiox14060649

**Published:** 2025-05-28

**Authors:** Adrián Báez-Ruiz, Andy Hernández-Abrego, Mauricio Díaz-Muñoz, Isabel Méndez

**Affiliations:** 1 Laboratorio de Ritmos Biológicos, Facultad de Ciencias, Universidad Autónoma de San Luis Potosí, Avenida Chapultepec 1570, Privadas del Pedregal, San Luis Potosí 78295, Mexico; adrian.baez@uaslp.mx; 2 Departamento de Neurobiología Celular y Molecular, Instituto de Neurobiología, Universidad Nacional Autónoma de México (UNAM), Campus UNAM-Juriquilla, Boulevard Juriquilla 3001, Querétaro 76230, Mexico; andy.bha@comunidad.unam.mx (A.H.-A.); mdiaz@comunidad.unam.mx (M.D.-M.)

**Keywords:** redox, metabolism, time-restricted feeding, food-anticipatory activity

## Abstract

Daytime-restricted feeding (TRF) exerts outstanding effects on circadian physiology, nutrient utilization, and energy metabolism. Limiting feeding access to two hours during the daytime (12:00–14:00 h) for three weeks promotes food-anticipatory activity (FAA). FAA encompasses not only behaviors related to meal expectations but also includes diurnal fluctuations in liver metabolic responses, including distinct redox handling. Hepatic microarray profiles of genes associated with redox response processes were analyzed at three crucial time points: at the beginning of the light period or before FAA (08:00 h), during the expression of FAA (11:00 h), and after feeding (14:00 h). Data on fasting and nutrient processing were integrated, whereas circadian implications were extrapolated by comparing the TRF transcriptional output with a one-day fasting group. Transcripts of redox reactions, such as reactive oxygen species (ROS) generation, antioxidant defenses, NAD^+^/NADH equilibrium, and glutathione, hydrogen peroxide (H_2_O_2_), arginine, nitric oxide (NO), and hydrogen sulfide (H_2_S) metabolism, were analyzed. Results showed a decline in antioxidant defenses at 08:00 h, followed by a burst of pro-oxidant reactions, preparation of glutathione metabolism factors, and a tendency to decrease H_2_O_2_ and increase NO and H_2_S during the FAA. Most of the findings observed during the FAA were absent in response to one-day fasting. Hence, TRF involves concerted and sequential responses in liver pro-oxidant and antioxidant reactions, facilitating a redox-related circadian control that optimizes the metabolic utilization of nutrients, which differs from a response to a simple fast-feed cycle.

## 1. Introduction

Every organism exhibits a functional connection between nutrient assimilation and processing and the metabolic landscape that allows an appropriate environmental adaptation. From this perspective, food quality, food availability, fasting and feeding intervals, and the timing of feeding are parameters that deeply influence the metabolic and physiological status [1]. In rodents, daytime-restricted feeding (TRF) protocols consist of limiting daily food intake to short periods for several consecutive days or weeks, which significantly impacts circadian physiology, including daily metabolic rhythms [2]. Particularly in rats, regular food access for two hours during the daytime results in a 40% calorie restriction [3], thereby promoting the expression of an alternative circadian oscillator known as the food-entrained oscillator (FEO). This oscillator is partially independent from the central pacemaker: the hypothalamic suprachiasmatic nucleus (SCN) [4]. Although the FEO has not been fully characterized, it is considered as an emergent physiological entity that measures biological time through the coordinated interaction among peripheral and central nervous system oscillators during TRF [5,6].

The anatomical location of the FEO remains unidentified, but research shows that the liver is crucial in the rhythmic, physiological, and biochemical responses associated with TRF because of its key role in nutrient metabolic processing of nutrients. Our group has focused on characterizing the metabolic and circadian hepatic adaptations as an entrainment response to TRF across different dimensions: (1) the circadian molecular clock [3], (2) mitochondrial and peroxisomal properties [7,8], (3) gluconeogenic activity and ureagenesis, carbohydrate, and lipid metabolism, [9,10,11,12], (4) and (5) detoxification actions [13].

Furthermore, rodents exhibit a distinct behavior under hypocaloric TRF, characterized by the emergence of locomotor activity preceding food access, known as food-anticipatory activity (FAA). This activity appears 2–3 h before meal availability [6,14] and persists for several days, even in the absence of food or other synchronizers. Moreover, it is accompanied by other hormonal and metabolic responses, including a peak in circulating glucocorticoids [15] and free fatty acids [16], and an oxidized hepatic redox state in both cytoplasmic and mitochondrial compartments [17]. Most of these events do not occur as direct reactions to a one-day fast (Fa). Interestingly, FAA occurs despite the experimental ablation of the SCN. Taken together, these findings support the notion that the FEO is an emergent adaptation in circadian physiology promoted by TRF [5,18]. In this context, we explore the possibility that TRF entrainment might involve redox fluctuations that could influence oxidative eustress in the hepatic tissue [19].

Food assimilation involves several metabolic processes that manage reactive oxygen species (ROS) and reactive nitrogen species (RNS), mainly in organs implicated in nutrient management, such as the liver [19]. Subtle levels of ROS/RNS participate in cellular signaling, promoting mitogenesis, immunological responses, and circadian modulation. In excess, these substances are capable of binding and irreversibly damaging lipids, proteins, or DNA [20]. In this respect, cells have antioxidant factors to counteract the excess of ROS/RNS. These include enzymatic pathways (i.e., catalase, superoxide dismutase, or glutathione peroxidase) and non-enzymatic factors (e.g., glutathione, vitamins, and metal ion carriers/chelating agents), respectively [20]. In this respect, it is relevant to mention that mitochondrial oxidative activity estimated by ATP synthesis occurring during the FAA exhibits a considerable increase, a situation that might considerably contribute to ROS emission [7]. Hence, a coordinated balance between ROS/RNS level production and antioxidant defenses could prevent oxidative stress, which is related to several pathological outcomes.

This work aimed to extend our understanding of liver adaptations in rats under TRF by characterizing the gene expression pattern associated with pro-oxidant and antioxidant responses through microarray analysis.

Metabolic pathways are dynamic entities that constantly regulate their substrate metabolites, the interacting crossroads, and the molecular outputs. Indeed, it has been postulated that fine control strategies are necessary to optimize the fluxes of energy and cellular components. In this context, redox homeodynamics has emerged as a principal regulatory scheme in metabolic control. Oxidative homeodynamics is characterized by continuous redox sensing, the activation of redox signal transduction, and the subsequent translation of these signals into the activation of multiple cellular stress responses [20].

This redox control requires biochemical switches that allow cells and organisms to respond to various stimuli, for example, growth factors, toxins, and changes in nutrient availability [21]. Hence, this work aimed to explore the transcriptional landscape associated with antioxidant responses and ROS and RNS management that accompanies the interesting metabolic challenge occurring in the liver during FEO expression through microarray analysis. The rationale of this approach was to explore potential changes in the activity of redox-regulated proteins (i.e., the redox proteome), which could influence the epigenetic metabolic landscape of the liver, as well as gene expression during FEO activity [20]. For this purpose, liver samples were taken at three strategic times of the FEO adaptations—before FAA (08:00 h), during FAA (11:00 h), and after FAA (14:00 h)—to infer the metabolic redox responses, and hypothesizing that because of the distinct metabolic status presented in these three conditions, different redox-related gene expression profiles will occur. Our data indicate sequential changes in the antioxidant/pro-oxidant response-related genes at all three time points, complementing a previous report that detected a mostly reduced lipoperoxidative activity in several hepatic endomembranes at these time points in rats subjected to a TRF schedule [22]. Additionally, we compared the effect of a single fast to distinguish between the effect of entrainment by TRF and the lack of food for one day in rats.

## 2. Materials and Methods

### 2.1. Animal Housing

Forty-two male adult Wistar rats (six weeks old; 200 ± 20 g body weight) were housed in acrylic cages (40 cm × 50 cm × 20 cm) in a 12:12 h light–dark photoperiod (lights on at 08:00 h, geographical time) and controlled room temperature (22 + 2 °C). Animals were given ad libitum access to water and normal food chow unless stated otherwise. Each experimental procedure was ratified and directed according to the institutional guide for the care and use of animals in biomedical research (Universidad Nacional Autónoma de México).

### 2.2. Experimental Protocol

**Figure 1:**
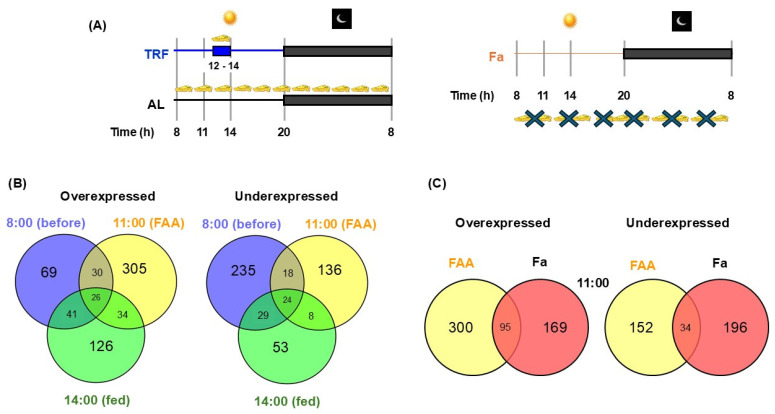
Experimental procedure and Venn diagram of microarray data analysis. (**A**) Schematic of the experimental protocols indicating the daily food access under ad libitum feeding (AL) or time-restricted feeding (TRF, food access for 2 h) for 3 weeks. One-day fasting (Fa) group was submitted to 21 h of fasting. The Venn diagram shows numbers of overexpressed and underexpressed genes in the liver of rats (**B**) under TRF at 08:00 h (before FAA), 11:00 h (FAA), and 14:00 h (fed rats), and (**C**) at 11:00 h under TRF, and Fa. The microarray analysis of every time point was performed relative to ad libitum conditions at the same time.

Once animals reached the expected age (6 weeks) and weight (200 ± 20 g), they were randomly sampled and assigned to each group by using the R software (V2.4) to one of three groups: control, TRF, and fasted. For the control group, eighteen rats were given ad libitum meal access. The TRF group consisted of eighteen rats fed daily from 12:00 h to 14:00 h (geographical time) for three weeks. The control and TRF groups were randomly separated into three subgroups of six rats each (Figure 1A). The rats were euthanized, and their livers were sampled at 08:00 h (before FAA), 11:00 h (FAA), and 14:00 h (after feeding). It was reported elsewhere that rats under this TRF protocol are thinner but without nutritional shortcomings [3,11,17]. The one-day fasted group (Fa) formed by six rats subjected to a single 21 h fasting period was euthanized at 11:00 h for liver sampling (Figure 1A). In addition, a single-blinded protocol was followed by samples, which were pooled and received a code to keep treatment assignments separate until after the correspondent assessment for microarray comparison.

### 2.3. Total RNA Preparation

Total RNA extraction with Trizol reagent was performed as described by Chomczynski and Sacchi [23]. For each of the control and experimental groups, 1 g of hepatic tissue sampled from five animals was used for total RNA preparation and subsequently pooled in aliquots to determine its purity, integrity, and concentration.

### 2.4. Printing of Arrays

*Rattus norvegicus* five thousand 50 mer oligo library from MWG-Biotech Oligo sets (High Point, Greensboro, NC, USA) were placed in 40 µM Micro Spotting solution (ArrayIt Brand Products, Sillicon Vallley, CA, USA). SuperAmine-coated slides measuring 25 mm × 75 mm (TeleChem International, Inc., Sunnyvale, CA, USA) were printed in duplicate and fixed at 80 °C for 4 h. For pre-hybridization, the slides were re-hydrated with water vapor at 60 °C and fixed with two cycles of UV light (1200 J). After boiling for two minutes, slides were washed with 95% ethanol for one minute and pre-hybridized in 5X SCS (sodium citrate-saline buffer), 0.1% SDS (sodium dodecyl sulfate), and 1% BSA (bovine serum albumin) for 1 h at 42 °C. The slides were washed and dried for further hybridization.

### 2.5. Probe Preparation and Hybridization for Arrays

An amount of 10 µg of total RNA was used for cDNA synthesis incorporating dUTP-Cy3 or dUTP-Cy5 from the CyScribe First-Strand cDNA labeling kit (Amersham, Amersham, UK). Fluorophore incorporation was confirmed by assaying the absorbance at 555 nm for Cy3 and 655 nm for Cy5, respectively. Equivalent quantities of labeled cDNA were hybridized using the hybridization solution UniHyb (TeleChem International, Inc., Sunnyvale, CA, USA) to the collection of “Rat Liver Arrays” for 14 h at 42 °C. For microarray determinations, a pool of total RNA from six animals corresponding to each condition previously detailed in the experimental protocol was used, and the fluorophore-associated signal was used as follows: dUTP-Cy3 for the control group, dUTP-Cy5 for the TRF group, and dUTP-Cy3 for the group of 11 h and dUTP-Cy5 for the Fa group, respectively.

### 2.6. Signal Acquisition and Data Analysis

Signal acquisition and quantification of array images were performed using ScanArray 4000 equipment and software version 2.0 (Packard BioChips) with 65–70% laser power and 60% gain. For each Cy3 or Cy5 fluorescence label, the average density and mean background were quantified with ArrayPro Analyzer software version 4.5 from Media Cybernetics (Bethesda, MD, USA). Microarray data analysis was carried out using genArise software (version 1.66), which was developed by the Computing Unit of the Cellular Physiology Institute at UNAM (CDMX, Mexico). This software mainly allows the correcting of fluorescence signals from the background and identifies transcripts exhibiting significant differential expression across experimental conditions. To achieve this objective, the z-score is calculated, where z represents the number of standard deviations a data point is from the mean. The transcripts with a z-score value greater (upregulated) or less (downregulated) than two standard deviations were considered differentially expressed. Then, a single experiment analyzing rat liver transcription was performed for each of the following comparisons: (1) before FAA (liver samples at 08:00 h from the control and TRF groups); (2) during the expression of FAA (liver samples at 11:00 h from the control and TRF groups); (3) after the FAA (liver samples at 14:00 h from the control and TRF groups) and (4) liver samples at 11:00 h from the TRF group and the Fa group. The set of genes was categorized by functional classification as members of gene families related to redox state using Gene Set Enrichment Analysis (GSEA, accessed on 13 March 2025 https://www.gsea-msigdb.org/gsea/index.jsp).

## 3. Results and Discussion

Daytime-restricted feeding (TRF) induces acute temporal nutrient optimization, which occurs at a specific time of day. In addition, TRF promotes a series of physiological adaptations, such as food anticipation, that facilitate the precise management of energy metabolism and other processes. Food-anticipatory activity (FAA) results from these adaptations evoked by TRF accompanied by minimum caloric intake. Therefore, metabolic and nutrient management processes are finely regulated around this time point. In addition, since energy status is directly related to redox metabolism, nutrient management processes are expected to exhibit specific alterations in this physiological context.

In this context, because of the predominant redox condition towards an oxidized state influenced by the TRF, this potentially leads to adjusting those redox homeostasis responses that are necessary to keep balance. At this respect, there are some master transcriptional factors which are responsible for upregulating antioxidant- and detoxification-related gene expression such as the case of Nrf2 (Nuclear Factor E2-Related Factor 2), NF-κb (Nuclear Factor-kappa B), HIF-1α (Hypoxia-Inducible Factor 1-alpha) or AP-1 (Activator Protein 1) [24]. Interestingly, *Nrf2* results with a downregulation at the TRF-8 h group in the present study, reinforcing the suggestion that a decrease in the induction of antioxidant-related genes occurs at this time point. Also, it has been reported that NF-Κb, an inflammatory and stress response-mediating protein, presents a peak just around the food restriction [25]. Finally, Nrf2, HIF-1, and AP-1 factors are redox-sensitive transcription factors that share a sensitivity to caloric restriction [24]. Although TRF protocol is not formally a strict caloric restriction protocol, it certainly involves a decrease in the amount of food consumed, therefore suggesting a potential influence in both HIF-1 and AP-1 factors, respectively.

Based on a liver microarray analysis (Figure 1B,C), transcripts with a score two-fold above the Z-score rate were classified as upregulated, and those below this reference index were classified as downregulated. Hence, Figure 1B illustrates that, in the comparison between TRF and ad libitum conditions, the TRF 11:00 h group (during FAA expression) exhibited a considerable upregulation of transcripts (305 genes), whereas the TRF 14:00 h group (fed group) demonstrated fewer downregulated transcripts. Interestingly, when comparing the TRF 11:00 h and Fa groups with the control group (Figure 1C), we found 300 transcripts upregulated in the TRF 11:00 h group and 196 upregulated transcripts in the Fa group. This result suggests that, although the TRF 11:00 h group is in a fasting state every day, it exhibits a distinct physiological profile when compared with a regular fasting state.

**Figure 2:**
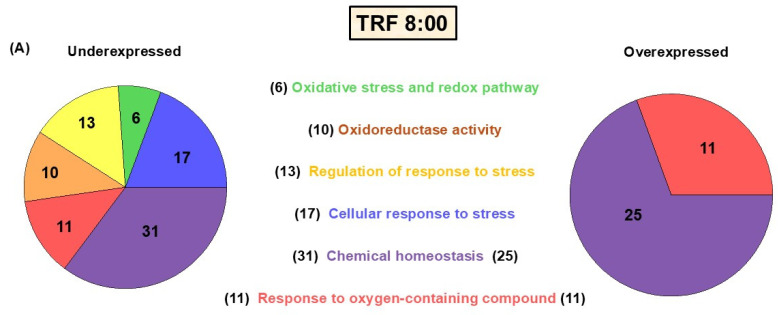

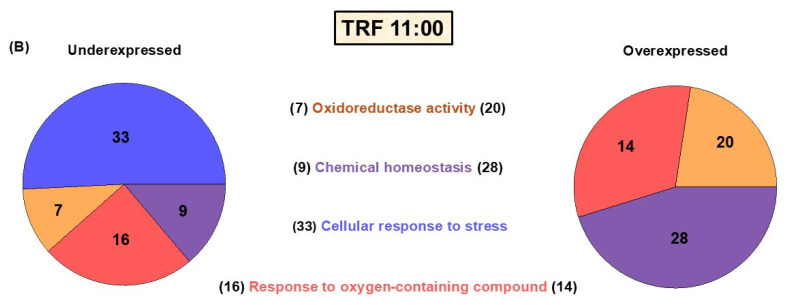

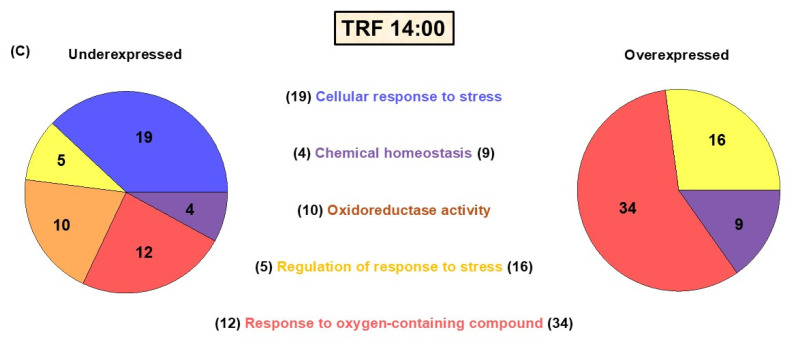

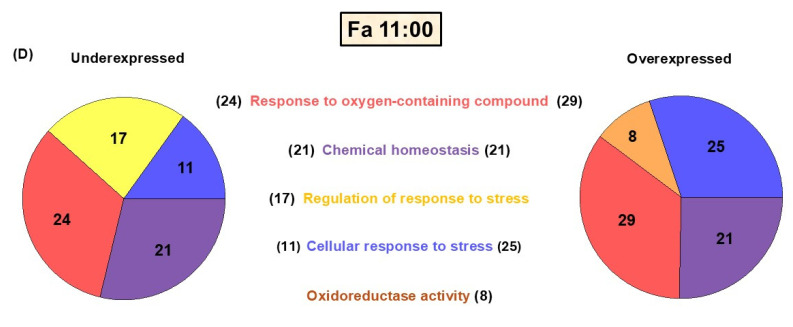
Categories of redox-related genes modified by TRF and Fa. Biological processes were obtained by GSEA analysis. Pie charts show the number of genes (shown in parentheses) that were underexpressed (left) or overexpressed (right) by at least two-fold. (**A**) 08:00 h, (**B**) 11:00 h, (**C**) 14:00 h under TRF, and (**D**) Fa at 11:00 h. The microarray analysis of every time point was performed relative to ad libitum conditions at the same time.

**Figure 3:**
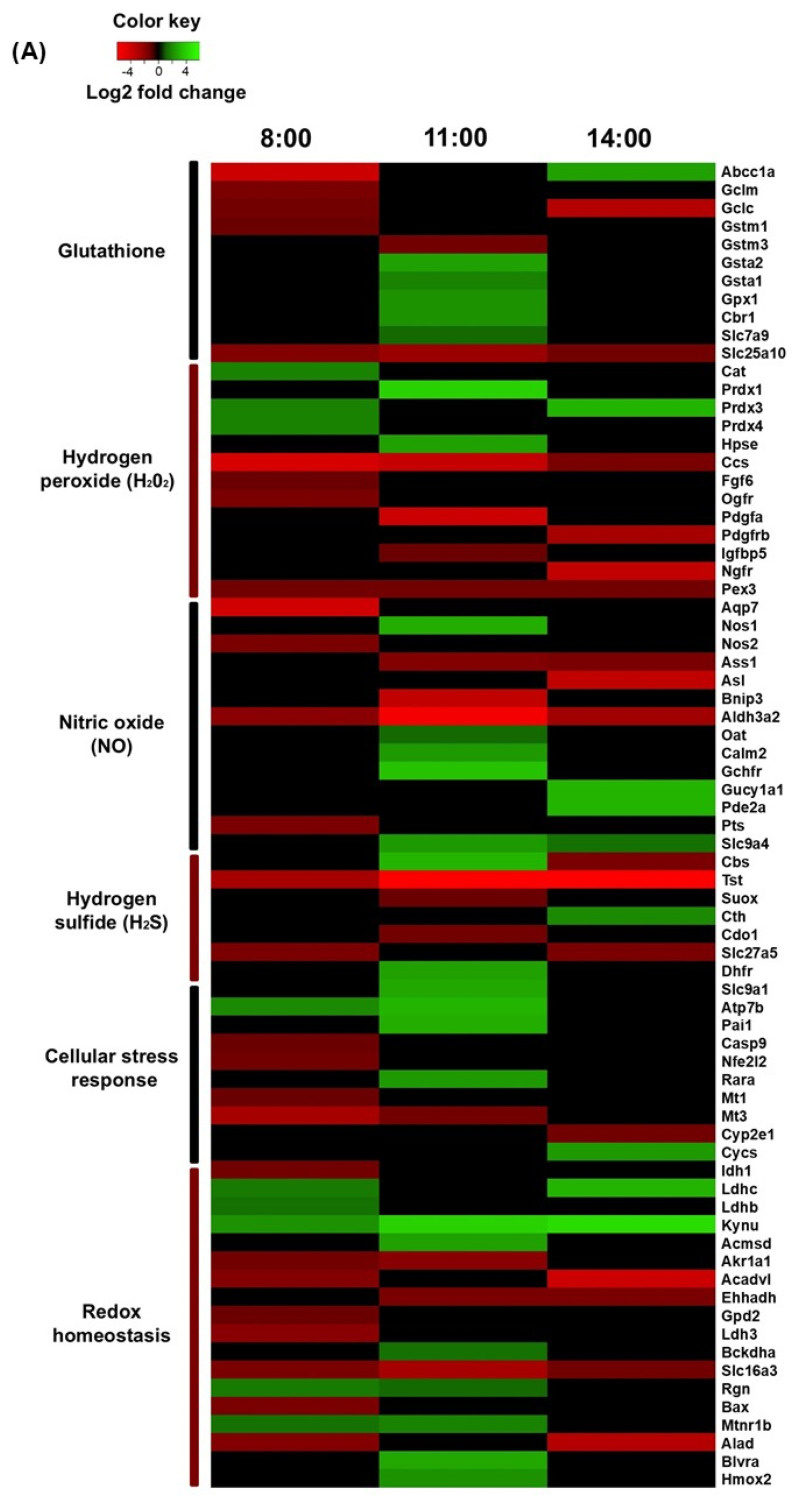

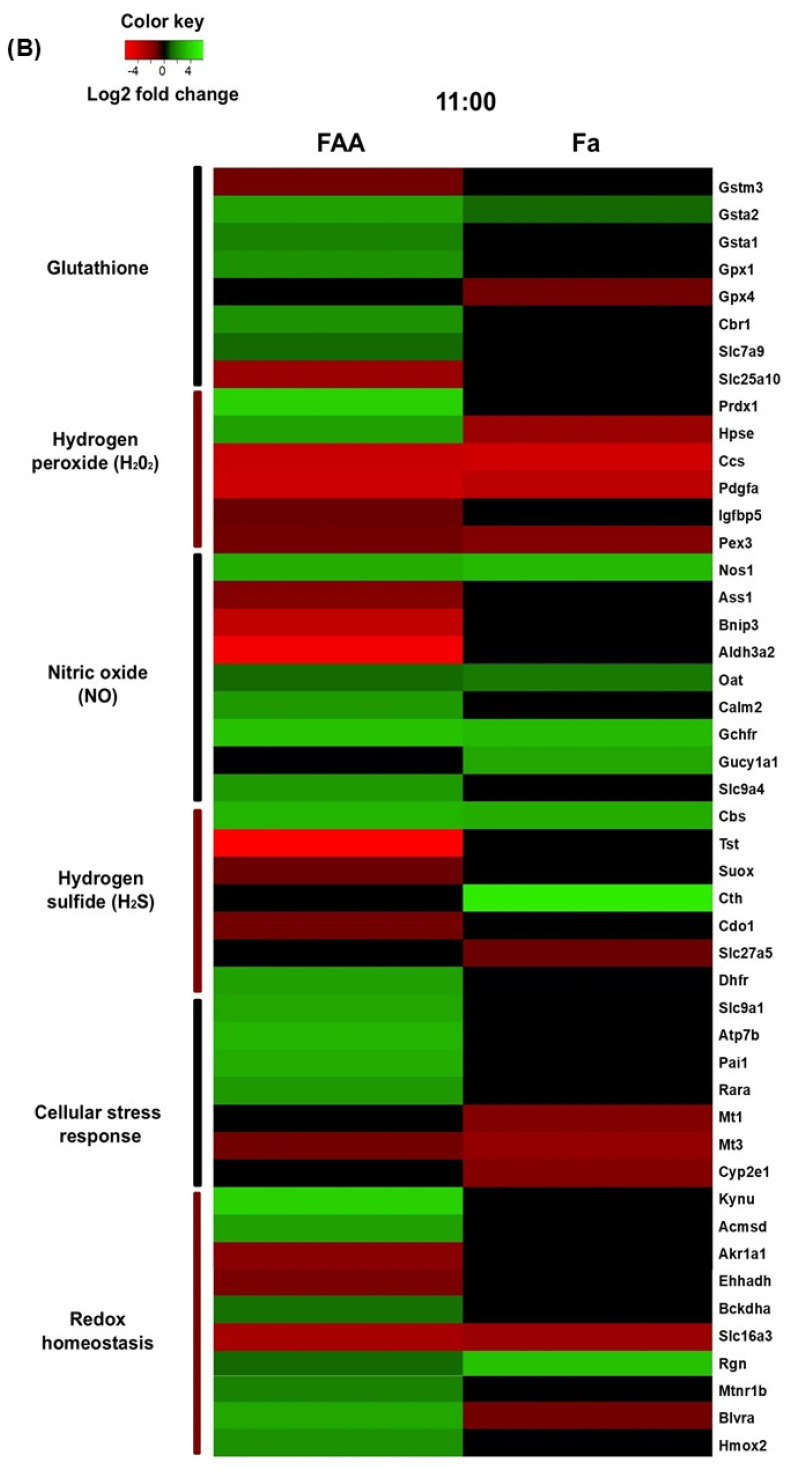
Heatmap of differentially expressed genes involved in the liver redox state (**A**) under TRF at 08:00 h, 11:00 h, and 14:00 h, and (**B**) under TRF and Fa at 11:00 h. All data are related to ad libitum conditions at the same time. The color scale indicates the magnitude of fold change (range = −4 to 4).

Seventy-four transcripts associated with redox homeostasis were found to significantly change their expression across the experimental group comparisons. The genes were subsequently ordered into groups based on the metabolic and/or signaling pathways associated with the proteins they encode (Figure 2A–D). The heatmap obtained from this analysis shows that most of the genes differentially expressed are down- or upregulated depending on the time point and the feeding protocol (Figure 3A,B).

### 3.1. Glutathione

**Figure 4:**
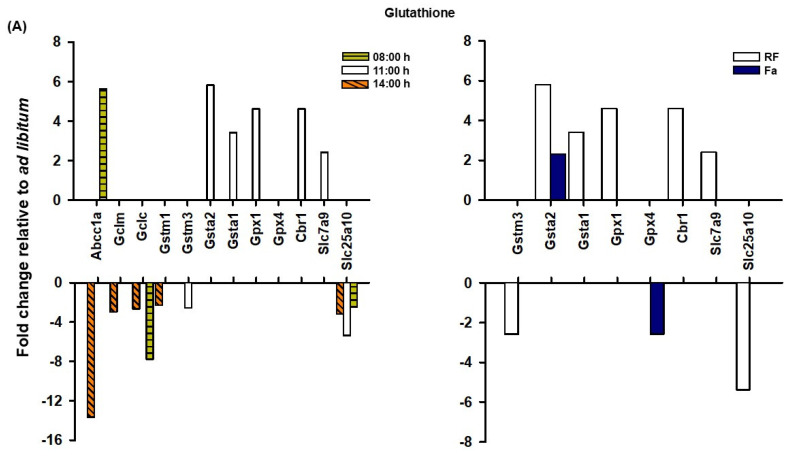

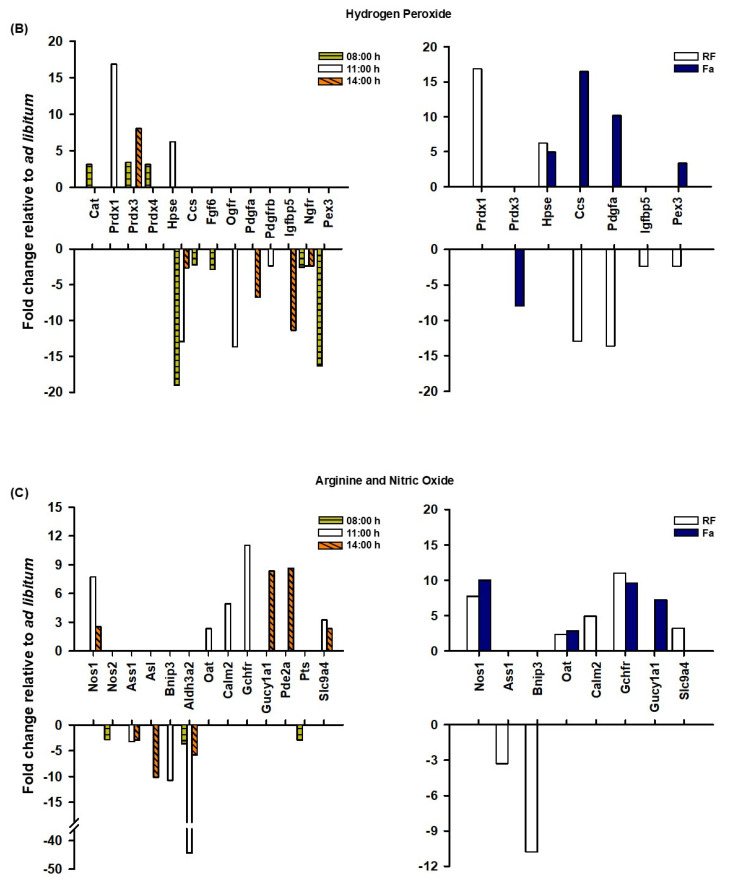

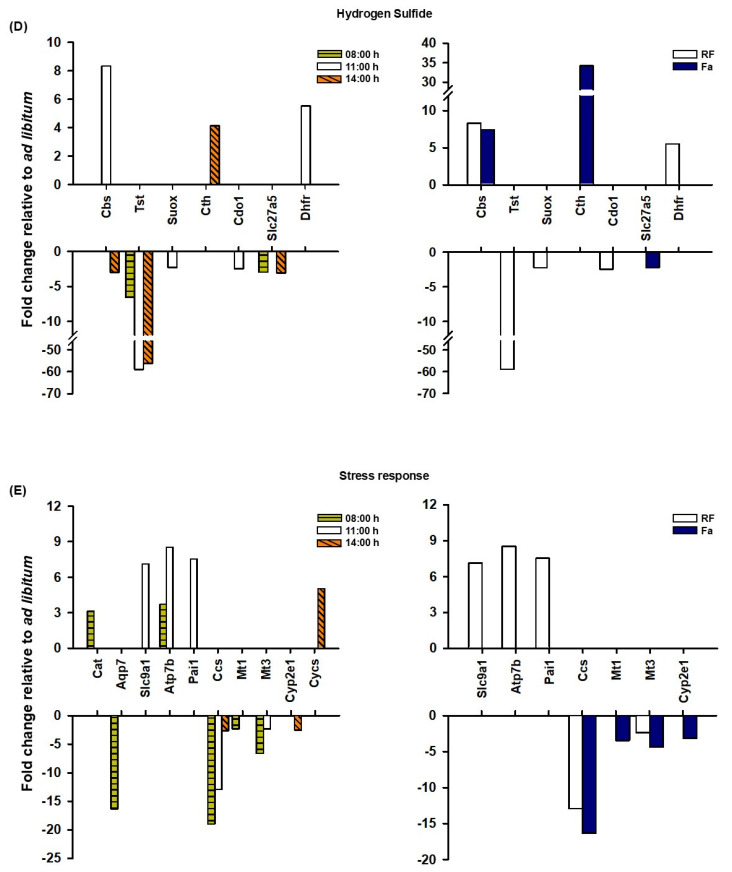

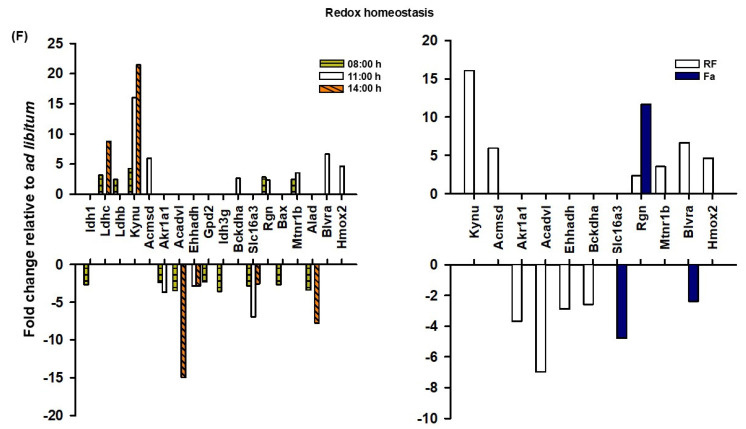
Differentially expressed genes of liver redox processes-related pathways: (**A**) glutathione, (**B**) hydrogen peroxide, (**C**) nitric oxide, (**D**) hydrogen sulfide, (**E**) stress response, and (**F**) redox homeostasis. Comparison of gene expression in terms of fold change as measured by microarrays based on data from Luna-Moreno, et al. 2007 [22]. Fold changes represent expression level of genes relative to ad libitum group. Comparison at 8:00 h, 11:00 h, and 14:00 h in TRF group (left panel), and comparison between TRF and Fa groups at 11:00 h (right panel).

Glutathione, or γ-glutamylcysteinyl glycine, is a tripeptide molecule with antioxidant properties that was discovered more than a century ago. It is considered one of the primary non-enzymatic antioxidant defenses in cells and is predominantly synthesized by the liver, despite its widespread presence [26]. In addition, this tripeptide molecule is not synthesized by ribosomes; rather, it requires a battery of enzymes (mainly glutamate–cysteine ligase and glutathione synthase), and amino acid carriers (specifically cationic amino acid transporters) to provide the raw materials: glutamate, cysteine, and glycine [26]. In this respect, we found a decrease in the modifier subunit glutamate cysteine ligase (*Gclm*) and in the glutathione synthase light chain (*Gclc*) in the TRF 08:00 h group. Additionally, the glutathione synthase light chain transcript exhibited an even more significant downregulation (7.8-fold) in the TRF fed group. However, the solute carrier family 7 (*Slc7a9*) (a cationic amino acid transporter for cysteine and glutamate) exhibited an increase in the TRF 11:00 h group during FAA expression. This finding suggested a decline in the production of this antioxidant element before or after FAA, along with an increase in the acquisition of substrates to biosynthesize glutathione during FAA (Figure 4A).

Complementarily, glutathione S-transferases (GSTs) are a diverse and abundant family of isoenzymes mainly produced by the liver. GSTs conjugate the reduced form of glutathione (GSH) into xenobiotic substrates for detoxification [27]. This process is known as phase II detoxification, which involves the removal of glutathione-conjugated molecules from the cell to prevent their accumulation. The TRF 11:00 h group presented an upregulation of two GST isoforms from the alpha family: glutathione S-transferase alpha 1 (*Gsta1*) (3.4-fold) and glutathione S-transferase alpha 2 (*Gsta2*) (5.8-fold) (Figure 4A). Interestingly, GST alpha family members are associated with the reduction of lipid peroxidation, which is typically upregulated in non-alcoholic fatty liver disease [28]. These results are significant as they indicate that an exacerbated oxidative lipid metabolism occurs in the liver during FAA [12]. Hence, metabolism related to FAA creates conditions conducive to severe oxidative stress, which might be alleviated by these antioxidant defenses.

Another group of enzymes, glutathione peroxidases, are responsible for reducing lipid hydroperoxides to alcohols and hydrogen peroxide (H_2_O_2_) to water [29]. Glutathione peroxidase 1 (*Gpx1*), the most abundant form, and glutathione peroxidase 4 (*Gpx*), which neutralizes lipid hydroperoxides, were upregulated in the TRF 11:00 h group. Additionally, carbonyl reductase 1 (*Cbr1*), another protective factor, exhibited a 4.6-fold increase in its expression during FAA. This enzyme reduces glutathionylated acetaldehydes derived from lipid peroxidation [30,31], which is consistent with the maintenance of defense patterns against exacerbated lipid metabolism occurring during the FAA (Figure 4A).

ATP-binding cassette subfamily C member 1 or ABCC1/MRP1 (*Abcc1a*) is a member of the ATP-binding cassette (ABC) transporter superfamily, functioning as an exporter that mediates the translocation of numerous substrates such as ions, sugars, amino acids, lipids, and drugs. It also cotransports its substrates with glutathione complexes, thereby regulating oxidative stress [32]. In addition to GSH and GSH complexes, MRP1 can transport glutathione disulfide (GSSG), the oxidized disulfide form of GSH. GSSG acts as an inhibitor of certain enzymes (e.g., adenylate cyclase) and an activator for others (e.g., aminolevulinate synthase). GSSG levels increase in cells under oxidative stress, and the efficient removal of this molecule with pro-oxidant activity is important for redox homeostasis [32]. The GSH/GSSG extruding pump presented a significant downregulation in the TRF 08:00 h group (13.7-fold with respect to the control group), whereas this pattern was reversed in the TRF 11:00 h group (during FAA expression), with a 5.6-fold upregulation compared to the control group. In contrast, the solute carrier family 25 member 10 (*Slc25a10*), a mitochondrial glutathione transporter essential for maintaining redox homeostasis [33], showed consistent downregulation across all time points in the TRF groups (Figure 4A). GSH levels are observed in both the cytosolic and mitochondrial compartments of the cell, with the latter being fundamental for cell survival [33]. This suggests that there are alternative antioxidant defenses for mitochondrial ROS production associated with FAA.

Hepatic glutathione levels in nocturnal animals exhibit a diurnal profile, peaking in the early morning during the post-absorptive state [34]. On the other hand, the nutritional state also impacts glutathione levels. Starvation for more than 24 h diminished glutathione levels by 50% or more, strongly suggesting that diurnal variation is related to the feeding regimen. In this respect, our results highlight a significant rheostatic management in the expression of genes associated with both the feeding condition and glutathione metabolism. The mitochondrial respiratory chain, along with mitochondrial and peroxisomal fatty acid oxidation, are the main cellular sources of ROS in the liver [35]. Previous studies by our group reported that hepatic mitochondrial activity, lipid oxidation, and peroxisomal biogenesis are significantly augmented during FAA [7,8]. Only during the FAA, mitochondrial oxidative activity, phosphorylating capacity, and NADH shuttles exhibit a significant increase, hence reflecting a potential ROS contribution. This condition suggests that a considerable increase in ROS levels is expected before meal intake, in association with the subsequent ROS production resulting from food consumption.

### 3.2. Hydrogen Peroxide

H_2_O_2_ is an important liver metabolite. Nearly 2% of the oxygen processed by the liver is directed towards H_2_O_2_. This compound is also a key factor in the cross-regulation between hormetic control over pro-oxidant reactions and its role as a second messenger for various paracrine signals [20]. Transcriptional data in this study strongly suggest that the liver’s metabolic profile downregulates H_2_O_2_ levels in response to two hours of daytime TRF (Figure 4B). We found an increased presence of peroxiredoxins or PRXs, H_2_O_2_-degradating enzymes, such as peroxiredoxin 1 (*Prdx1*), 3 (*Prdx3*) and 4 (*Prdx4*), glutathione peroxidase 1 (*Gpx1*) and 4 (*Gpx4*), and peroxisomal catalase (*Cat*), at the three time points studied. Furthermore, transcriptional expression of peroxisomal biogenesis factor 3 or PEX3 (*Pex3*) is significantly reduced at 08:00, 11:00, and 14:00 h, further strengthening the notion that catalase and peroxisomal activity are important during FEO expression in the liver [8]. In 2014, Yamashita et al. reported that the membrane protein PEX3 promotes peroxisomal autophagic destruction [36]. Hence, the diminution of PEX3 would indicate a more relevant role for hepatic peroxisomes in the expression of FEO. Interestingly, these H_2_O_2_-degradating enzymes are present in both cytoplasmic and mitochondrial compartments. At 08:00 h, we observed a generalized reduction in the hepatic antioxidant outline, making these enzymes more noticeable. Hence, the prevailing pattern is that low levels of H_2_O_2_ are accompanied by reduced synthesis of proteins that transport H_2_O_2_, such as aquaporin 7 (*Aqp7*). This tendency was supported by the observation that several growth factors and a set of their receptors, which are known to produce H_2_O_2_ upon activation, were also downregulated at all three time points studied. Additionally, the expression of heparinase (*Hpse*), a protein that limits the availability of growth factor ligands, was enhanced at 11:00 h (Figure 4B).

Our data did not reveal changes in the transcriptional status of superoxide dismutases or SODs and nicotinamide adenine dinucleotide phosphate oxidases or NOXs which are key generators of H_2_O_2_. However, the copper chaperone for SOD (*Ccs*) tested three times in this study were significantly reduced, suggesting that the synthesis of Cu^2+^-Zn^2+^ cytoplasmic SOD1 was inhibited before, during, and after FAA (Figure 4B). Consistent with this finding, we observed an increase in regucalcin (*Rgn*) at 08:00 h and 11:00 h under TRF (Figure 4F), and an important elevation in response to Fa. It has been reported that this intracellular Ca^2+^ binding protein is upregulated in transgenic rodents lacking SOD1 expression [37].

John S. O’Neill and Akhilesh B. Reddy initially reported the existence of a non-transcriptional circadian metabolic clock in red blood cells [38] but later confirmed its presence in a variety of organisms including cyanophytes, fungi, plants, and mammals [39]. A central element of this metabolic/redox circadian clock was PRXs, one of the most effective catabolic enzymes for H_2_O_2_. The redox activity of this timing system was monitored by the 24 h rhythmicity of the sulfenic and sulfinic forms of PRX in a system that depends on NADPH availability and thioredoxin activity. In 2012, Edgar and colleagues observed a rhythm in PRX1 within the mouse liver [39]. The peak of this enzyme was out of phase with the peak of clock-gene Bmal1. In this study, we did not measure the redox state of PRXs, but we found that that, in addition to *Prx1*, *Prx3,* and *Prx4* also exhibited upregulation before and after feeding (Figure 4B). Interestingly, a discrepancy between the TRF-11h and the fasted groups was also observed: while the *Prdx1* and Heparanase genes denoted an upregulation in the TRF-11 h group, the copper chaperone for SOD, Platelet-derived growth factor A chain (*Pdgfa*) and Pex3 resulted with an upregulation in the Fa group, respectively (Figure 4B). This accentuates the distinct redox responses occurring in the TRF-11 h group in terms of the H_2_O_2_ management.

In summary, our transcriptional data strongly suggest that hepatic tissue may exhibit low levels of H_2_O_2_ before, during, and after FAA. The potential decrease in this oxidant molecule seems to be accompanied by a diminished antioxidant response at 08:00 h and a pro-oxidant activation involving GSH-dependent reactions at 11:00 h. Overall, these data could serve to undertake a new working hypothesis regarding the role of H_2_O_2_ as second messenger in the context of hepatic circadian physiology.

### 3.3. Arginine Metabolism and NO Signaling

Nitric oxide (NO) is a free radical and volatile messenger that is synthesized from the amino acid L-arginine by a set of three isoenzymes: neuronal NO synthetase or NOS1, inducible NOS or NOS2, and epithelial NOS or NOS3. NO is considered an RNS by itself. At discreet concentrations, the most usual signaling pathway for NO is by activating the membranal and soluble form of guanylate cyclase to form cGMP; then, the cyclic nucleotide activates protein kinase G (PKG), which phosphorylates multiple substrates. However, NO at high levels can react with diverse biomolecules to favor nitrosative signaling or a stress response [40].

NO generation involves the parallel synthesis of citrulline in a redox reaction that also requires biopterin and NADPH. In the liver, L-arginine is a common substrate for NOS and arginase (urea synthesis in periportal hepatocytes). Therefore, the prevalent activity should be finely regulated even by selective metabolic activation/inhibition or by hepatic zonation that allows differential expression and distribution according to the type of hepatocyte (periportal/pericentral) [41]. Luna-Moreno and colleagues reported in 2012 that circulating urea levels exhibited a dramatic diminution in rats subjected to TRF after food access [9]. This fluctuation in urea levels seems to correlate with the transcriptional data in this study, in which a reduction in NOS2 was detected at 08:00 h and arginosuccinate synthetase (*Ass1*) at 11:00 h and 14:00 h, as well as a decrease in arginosuccinate lyase (*Asl*) at 14:00 h (Figure 4C). These data suggest that in rats subjected to TRF, urea synthesis occurs before FAA, at 08:00 h, and that at 11:00 h, during FAA, there is a metabolic switch that suppresses urea formation and favors NO formation. This last point is supported by the transcriptional increase in NOS1 (*Nos1*) at 11:00 h and 14:00 h, as well as the enhancement of transcripts for calmodulin 2 (*Calm2*), an activator of NOS1, at 11:00 h and for the soluble form of guanylate cyclase (*Gucy1a1*) at 14:00 h (Figure 4C). In addition, at 11:00 h we observed an increase in GTP cyclohydrolase (*Gchfr*), a key enzyme in the synthesis of tetrahydropterin, which acts as a cofactor for NOS activity [42].

It is appropriate to note that although NOS1 and NOS2 catalyze the same reaction, NO formation, there are differences in their expression in the liver. For example, NOS1 is usually constitutive in hepatic cells producing a small quantity of NO, while NOS2 produces larger amounts of NO for longer intervals, serving as a significant effector during inflammation and infection [43]. NOS2 is primarily found in periportal hepatocytes and also Kupffer cells [44]; in contrast, NOS1 is located in pericentral hepatocytes, as well as in endothelial and Kupffer cells [45].

Hence, the urea cycle and production of NO can be observed in the metabolic profile of rats under a TRF protocol before and during FAA, as well as after feeding. This highly suggests that there is a biochemical adaptation in the differential use of L-arginine. At 08:00 h, before FAA, periportal hepatocytes are engaged in urea formation with transcriptional suppression of NOS2; at 11:00 h, during the FAA, arginine is directed towards NO synthesis by NOS1 in pericentral hepatocytes, which leads to PKG activation and molecular target phosphorylation. Subtle differences were demonstrated in comparison with the Fa group respecting arginine metabolism, since the TRF-11 h showed a decreased expression of the arginosuccinate synthase 1 (Figure 4C). It is also possible that the formation of the pro-oxidative molecule peroxynitrite is not favored, since the hepatic system at those time points expresses catabolic enzymes for H_2_O_2_, and subcellular peroxidative activity in the liver is significantly reduced [22], indicating that the presence of superoxide is unlikely.

### 3.4. Hydrogen Sulfide and Sulfur-Containing Metabolites

Redox regulation based on sulfur-containing metabolites is complex and diverse. It involves mainly reversible oxidative changes in reactive cysteines but also includes the metabolic participation of methionine-related intermediates [46]. In addition to the glutathione system and proteins/enzymes with reactive cysteine residues and transition metal centers with sulfur ligands, hydrogen sulfide (H_2_S) plays a central role in redox biology.

H_2_S is a gaseous molecule first recognized in 1996 as an endogenous regulatory factor in intermediate metabolism [47]. Today, it is considered a ubiquitous redox regulator with a preponderant role in mitochondrial function [31]. H_2_S is synthesized by the coordinated activity of three enzymes: cystathionine β-synthase, cystathionine γ-lyase, and 3-mercaptopyruvate sulfurtransferase. The level of H_2_S in each tissue results from its formation and oxidation fluxes. Its catabolic processing involves several mitochondrial enzymes: sulfide: quinone oxidoreductase, rhodanese/thiosulfate transferase, sulfur dioxygenase, and sulfite oxidase.

The transcriptional analysis of hepatic tissue during FEO expression strongly suggests an increased presence of H_2_S at the three time points studied, particularly during FAA (11:00 h), before food access. Remarkably, we observed a substantial decrease in the rhodanese (*Tst*) transcript at 08:00, 11:00, and 14:00 h without changes in Fa group (Figure 4D). The values obtained for this catabolic factor at 11:00 h and 14:00 h under TRF were markedly low (59.10 and 56.25, respectively). Carter et al. (2021) [48] reported high levels of circulating and hepatic H_2_S in a *Tst*-deficient mouse model, as well as activated gluconeogenesis and ameliorated liver fatty acid oxidation. A transcript for the catabolic enzyme sulfite oxidase (*Suox*) also presented a reduction at 11:00 h. Our results showed an increase in the transcripts of H_2_S-forming enzymes cystathionine β-synthase (*Cbs*) at 11:00 h while cystathionine γ-lyase (*Cth*) at 14:00 h, supporting this trend (Figure 4D). Both transcripts were found to be upregulated in response to fasting, indicating a response to the fasting state (Figure 4D). Cystathionine synthesis for H_2_S production requires cysteine availability, a condition that is further favored by the reduction in cysteine dioxygenase (*Cdo1*) transcript levels at 11:00 h, leading to the inhibition of taurine synthesis. Taurine is a metabolite involved in the formation of bile acids [49]. The diminution of bile acid coenzyme A ligase (*Slc27a5*), which attaches taurine to the bile acid molecule, also contributes to this possibility. Finally, another potential substrate for cystathionine formation is homocysteine. Homocysteine is strongly related to methylation reactions that depend on S-adenosylmethionine (SAM); after a methyl transfer reaction, SAM is transformed into SAH (S-adenosylhomocysteine). SAH is split into adenosine and homocysteine. At 11:00 h, the dihydrofolate reductase (*Dhfr*) transcript was elevated solely in response to TRF, while it showed not responsive to Fa; therefore, this elevation indicates an entrainment response due to timely food access. This enzyme is essential in the recovery of methionine to restart the methylation cycle [46].

### 3.5. Stress Response

The coordinated regulation of several genes ensures liver redox balance by regulating detoxification, metal ion homeostasis, and cell survival among other functions. In this regard, members of the Slc family, such as solute carrier family 9 member A1 (*Slc9a1*) that encodes to sodium/hydrogen exchanger, regulates the fine control of intracellular pH and ion homeostasis, ATPase copper transporting beta (*Atp7b*) that encodes a copper-transporting ATPase are essential for the prevention of oxidative damage, and Serpine1 (*Pai1*) which controls extracellular matrix maintaining the tissue homeostasis and is upregulated in response to inflammatory and oxidative signals [50,51,52], all of them are upregulated during the FAA (Figure 4E). Also, retinoic acid receptor alpha (*Rara*) plays a critical role promoting antioxidant actions on iron-induced oxidative stress in the liver [53] and displays a higher expression at FAA comparing to ad libitum conditions.

On the other hand, metallothioneins are small, cysteine-rich proteins that bind heavy metals and exert key cell protective actions against oxidative stress. In the liver homeostasis, metallothionein 1 (*Mt1*) and metallothionein 3 (*Mt3*) have an important control of the hepatic metal detoxification [54]. Both genes are downregulated at 8:00 h and Mt3 at 11:00 h in TRF, which could contribute to an oxidized environment early before food access. It seems to be a response to entrainment because the response between TRF and Fa can be distinguishable, at the same temporal point (Figure 4E).

Cytochromes are known to participate importantly in the liver redox regulation. It is the case of cytochrome P450, family 2, subfamily e, polypeptide 1 (*Cyp2e1*) which induces the production of ROS and acetaldehyde from ethanol metabolism causing cellular toxicity [55]. The TRF downregulates the expression of this gene after food access and is differentially regulated by Fa (Figure 4E), while cytochrome c, somatic (*Cycs*) is upregulated after food in the TRF group, but not in Fa. In the intrinsic pathway of apoptosis, cytochrome c allows apoptosome formation and caspase 9 activation which activates caspase 3 and caspase 7 leading to cell death [56]. Caspase 9 is activated in response to oxidative stress and inflammation, and its gene *Casp9* is downregulated at 8:00 h under TRF, which is consistent with the absence of inflammation and stress [25]. These data evidence the differential gene expression response to stress environments to TRF before and after meal and are divergent to Fa. These are examples of genes that respond to oxidative stress or situations that cause metabolic imbalances in liver function regulation. Therefore, their fine regulation in physiological conditions, such as circadian rhythmicity, is essential for cellular adaptation to environmental challenges for maintaining liver homeostasis.

### 3.6. Redox Homeostasis: NAD(P)^+^/NAD(P)H

The NAD^+^/NADH ratio is a prototypic metabolic regulator in the balance between anabolic and catabolic pathways. Controlled electron fluxes dictate the availability of reduced or oxidized coenzymes that facilitate the redox balance of fluctuating intermediate metabolites in circadian variations and nutrient processing [57]. In this regulatory concert, two parameters are crucial from a metabolic perspective: (1) the expression of enzymes capable of transferring electrons or hydrogen atoms, mainly dehydrogenases, and (2) the synthesis of the NAD^+^ molecule from tryptophan-related metabolites [58]. Indeed, researchers have reported a circadian modulation of the NAD^+^-biosynthetic enzyme nicotinamide phosphoribosyl transferase [59].

Previously, we reported that TRF schedules promoted and oxidized a cytoplasmic and mitochondrial redox state before food access at 08:00 h and 11:00 h, which was subsequently reversed upon feeding. The magnitude of this response differed from that observed after a 24 h fasting period [17]. Many transcripts in this group displayed expression changes in response to the synchronizing effect of the TRF regimen, but not to Fa condition. The analysis of transcripts in this study seems to support the nutrient-related NAD^+^/NADH ratio oscillation in response to TRF. Specifically, two anabolic enzymes in NAD^+^ formation were upregulated: kynureninase (*Kynu*) and 2-amino-3 carboxy-muconate 6-semialdehyde decarboxylase (*Acmsd*) (Figure 4F). Kynureninase transcript levels were elevated at the three time points, with particularly high values at 11:00 h (16.0) and 14:00 h (21.4); the other enzyme was elevated during FAA (5.9 at 11:00 h). These changes suggest an adaptation of NAD^+^ metabolism during TRF to an oxidized state. High levels of pyruvate before food access are correlated with the upregulation of two lactate dehydrogenase (LDH) isoforms—lactate dehydrogenase B (*Ldhb*) and lactate dehydrogenase C (*Ldhc*) at 08:00 h (2.4 and 3.1, respectively). LDHB favors the conversion of lactate to pyruvate when lactate is directed toward mitochondrial metabolism [60]. In contrast, LDHC has a glycolytic and ATP-synthetizing role in sperm flagella [61] but its hepatic function is not well understood [62]. A parallel result related to liver lactate and pyruvate was the downregulation of solute carrier protein 16a3 or MCT3 (*Slc16a3*), at all three time points, with a more accentuated reduction at 11:00 h both under TRF and Fa (Figure 4F), indicating a response relative to the fasting state. This was one of the few genes that also exhibited a response to Fa. This monocarboxylate carrier transports lactate and pyruvate across cellular membranes, possibly including those of mitochondria and peroxisomes [63]. The generalized reduction in MCT3 may be associated with a decrease in the mobilization of lactate and pyruvate across subcellular compartments due to the TRF protocol.

Several transcripts for NAD(P)^+^-dependent enzymes were downregulated in the livers of rats subjected to the TRF protocol, specifically Acyl-CoA dehydrogenase (8:00 and 14:00 h) and enoyl-CoA dehydrogenase (*Acadvl*) (at 11:00 h and 14:00 h) (Figure 4F). These changes are consistent with previous studies showing a peak in hepatic mitochondrial and peroxisomal β-oxidation at the middle of the light phase at 17:00 h, with no changes at 11:00 h and 14:00 h [12]. Additionally, these studies noted a peak in glycerol 3-phospate dehydrogenase (*Gpd2*) at 08:00 h, which converts the glycolytic intermediate dihydroxyacetone phosphate into glycerol. Two forms of isocitrate dehydrogenase were also observed downregulated at 8:00 h, one mitochondrial from the Krebs cycle, isocitrate dehydrogenase (NAD(+)) 3 non-catalytic subunit gamma (*Idh3g*), and the other cytoplasmic, isocitrate dehydrogenase (NADP(+)) 1 (*Idh1*) (Figure 4F). It is important to highlight that isocitrate dehydrogenase 1 is an NADP^+^-dependent enzyme and plays a role in the antioxidant defense system. This further exemplifies the tendency observed in our study, where antioxidant defenses were reduced in the liver of TRF rats at 08:00 h. In contrast, one mitochondrial dehydrogenase was upregulated at 11:00 h (Figure 4F), the E1 subunit of branched-chain α-ketoacid dehydrogenase (*Bckdha*), which is a catabolic enzyme for leucine, valine, and isoleucine. This enzyme converts NAD^+^, elevated in the hepatic mitochondria of TRF rats [17], into NADH.

Another aspect of ROS-related homeostasis is the redox processing of aldehyde intermediates resulting from lipoperoxidative activity. Lipid peroxidation involves an oxidative fragmentation of unsaturated acyl moieties within membrane phospholipids, leading to the formation of various aldehydic molecules. These aldehydes can be reduced to alcohol or oxidized to acid according to the redox drive of the cellular compartment. Previous observations from our research group indicated a reduction in the pro-oxidant reactions in the liver of TRF rats measured as the presence of conjugated dienes and TBARS response before, during, and after FAA [22]. This is consistent with the reduction in the aldehyde dehydrogenase 3A2 (*Aldh3a2*) transcript at the three time points tested in this study. Aldehyde dehydrogenase 3A2 is the enzyme responsible for the Sjögren–Larsson syndrome [64].

### 3.7. Influence of TRF on Clock Genes Expression and Redox Status

Single fasting or acute fasting (only one-day fasting) triggers hormonal and metabolic cellular responses. These responses enable metabolic reactions to provide energy sources to the organism. TRF is a regimen that involves repeated cycles of fasting–feeding every day, which has implications for circadian rhythms. Unlike a single fasting, the TRF regimen acts as a potent zeitgeber influencing the peripheral expression of core circadian clocks, particularly in the liver, including Bmal1, Clock, Per1/2, and Cry1/2, that can be observed over the amplitude and acrophase of their characteristic rhythm [3,65]. These changes on clock gene expression are part of the adaptations displayed by organs such as the liver that have metabolic actions as a primary function [5] with a wide physiological consequences, for example, redox homeostasis. It is clear that loss of synchronization between molecular clock and NAD+/NADH balance regulates metabolism [66]. The effects of TRF include changes in rhythmic patterns of NAD+ involving sirtuins, key regulators of redox balance [67], as well as transcriptional-independent mechanisms that involve peroxiredoxin oxidation cycles influenced by the availability of NADPH [68].

### 3.8. Limitations and Shortcomings

There are several considerations related to the experimental approach of this study that should be noted as opportunities to broaden future perspective regarding the transcriptional activity of the liver during FEO expression. For example, the data of this study were transcriptional, but analyzed and integrated with the perspective of several sets of metabolic results from previous reports. Hence, many of the proposals made in this project can serve as an informative hypothesis for subsequent experiments with the metabolites and enzymes mentioned in Section 3. Eventually, an analysis covering a complete daily time-series by using RNA-seq might represent other complementary aspects that by the nature of the present experimental design are out of sight. It is also pertinent to mention that it is usual to validate changes in determined transcript expression by qPCR. However, due to the exploratory approach of the present analysis, it would be difficult to rule out which genes were more important than others, from the approximately 74 transcripts that were found with changes in their expression profile. In addition, the present report explored the mRNAs of genes associated with redox reactions in liver sample homogenates; hence, it is not possible to define the hepatic cell type that originated the hepatic transcripts. Within the liver, there are parenchymal (hepatocytes) and non-parenchymal cell types (stellate, Kupffer, and endothelial cells, as well as cholangiocytes); in addition, hepatocytes are zonated, meaning that they display different metabolic landscapes according to their anatomical position in the hepatic lobule (periportal and pericentral hepatocytes) [69]. Therefore, transcriptional activity in periportal hepatocytes (more oxidative milieu) is anticipated to differ from that of pericentral hepatocytes (less oxidative milieu) [70]. We believe our current findings are significant because they highlight important transcriptional changes at the tissue level, suggesting a need for further investigation at the cellular level. To better understand the cell-specific mechanisms driving these transcriptomic changes, additional methods such as single-cell RNA sequencing could provide valuable complementary information. Another important limitation is related to the fact that pro-oxidant and antioxidant balance varies across subcellular compartments. To support this concept, ref. [71] reported that enhancement of lipid peroxidation occurred mainly in plasma membrane and cytosolic fractions during partial hepatectomy, whereas in acute CCl_4_ administration, it was observed in microsomal and nuclear membranes. This consideration was tested through a protocol involving TRF for 2 h during the daytime, since it was reported that differential lipoperoxidative activity and the presence of conjugated dienes were observed in subcellular fractions of liver homogenate before, during, and after FAA [22]. In addition, we acknowledge that our study provides a comprehensive view of redox-related gene expression changes in the male liver, establishing a reference point. In this respect, sexual dimorphism has been observed in female cells’ response to oxidative stress, the effect of sex hormones on redox state regulation, and the differential management of redox responses by various organs [72]. This highlights female circadian physiology as a promising area for further exploration. Finally, since TRF schedules have been used as an experimental protocol to influence circadian physiology [4,5], it would be interesting to extend this study to explore the 24 h rhythmicity of transcriptional activity in the liver during the expression of the FEO. TRF without a sustained hypocaloric restriction is a non-pharmacological and low-cost intervention for metabolic pathologies. Implementation of an individual regimen of TRF could be a chronobiological strategy for health optimization through redox management.

## 4. Conclusions

Our data confirms the complexity associated with the expression of the elusive FEOs. TRF deeply influences multiple parameters, such as foraging behavior, digestive physiology, and the timing system, through mechanisms that coordinate various metabolic pathways among cellular types, tissues, and organs. Our findings on the hepatic transcriptional profile associated with redox metabolic activity clearly show a differential outline depending on the times at which FAA is displayed. A striking finding was the concerted reduction in antioxidant factors at 08:00 h, before FAA. This result is consistent with a report in which an increase in TBARS and conjugated dienes in several liver subcellular fractions in rats expressing the FEO were detected at that time [22]. Another relevant finding was observed at 11:00 h, during the FAA; at this time, a marked proportion of transcripts were related to the formation of glutathione, NO, H2S, and enzymes that catabolize H_2_O_2_. And no less important is that the 14:00 h TRF group exhibits a reduced redox response in comparison to the 11:00 h TRF group, even though it was already fed. Interestingly, most of these changes were privative of livers of TRF rats and not detected in samples from the one-day fasting group, indicating that the metabolic adaptations during FAA were different to the adjustments in a single fasting event. Many of the results obtained in this study are convenient to be reinforced with biochemical and cellular approaches. Overall, the transcriptional profile of liver samples from rats under TRF schedules offers an initial perspective regarding the complex regulation involved in the metabolic and circadian control that facilitates the expression of hepatic FEO.

## 5. Perspectives

There is a straightforward connection between feeding and metabolism. In this context, TRF, as a modality of intermittent fasting, is an experimental maneuver that allows us to ask direct molecular and biochemical questions about the dynamic relationship of metabolic networks and circadian physiology. A natural consequence of this complexity is the link between pathological outcomes such as overweight, hypertension, inflammation, dyslipidemia, diabetic predisposition, and aging with an altered circadian molecular clock. Certainly, more basic research opportunities as well as translational applications based on nutritional protocols await to be approached in the near future.

## Data Availability

Authors declare that data were analyzed for this manuscript and has not been included in any repository or publicly archived dataset.
